# Brain Structural Covariance Networks in Long-Term First-Person Shooter and Multiplayer Online Battle Arena Players: Cross-Sectional Study

**DOI:** 10.2196/79976

**Published:** 2026-05-04

**Authors:** Zhenggen Lin, Fujia Jiao, Yuanbo Ma, Jie Zhuang, Yu Liu

**Affiliations:** 1 Key Laboratory of Exercise and Health Sciences of Ministry of Education, Shanghai University of Sport, Shanghai, China; 2 School of Exercise and Health, Shanghai University of Sport, Shanghai, China; 3 Department of Psychology, School of Humanities and Social Sciences, University of Science and Technology of China, Hefei, Anhui, China; 4 School of Sports and Health, Shenyang Sport University, Shenyang, Liaoning, China; 5 Department Psychology and Neurosciences, Leibniz Research Centre for Working Environment and Human Factors, Dortmund, Nordrhein-Westfalen, Germany; 6 Department of Psychology, Ruhr University Bochum, Dortmund, Nordrhein-Westfalen, Germany; 7 School of Psychology, Shanghai University of Sport, Shanghai, China; 8 School of Physical Education, Guizhou University of Engineering Science, 1 Xueyuan Road, Qixinguan District, Bijie, Guizhou, 551700, China

**Keywords:** video games, auditory attention, cortical thickness, individualized structural covariance network, network-based statistic prediction, support vector machine

## Abstract

**Background:**

The relationship between video game experience and cognitive plasticity remains a central focus of research, particularly given its potential applications in clinical rehabilitation. Although both first-person shooter (FPS) and multiplayer online battle arena (MOBA) games have been shown to enhance cognitive functions, the specific associations between the cognitive effects of different game genres and brain network structure remain unclear.

**Objective:**

This study aimed to examine whether long-term experience with FPS and MOBA games is associated with genre-specific patterns of cortical thickness covariation across brain regions.

**Methods:**

A total of 116 male participants (mean age 21.2, SD 1.9 y) were recruited via online advertisements for this cross-sectional study. On the basis of strict inclusion criteria (gaming experience >5 years, gaming frequency >5 hours per week, and ranking within the top 15%), participants were categorized into FPS players (n=39, 33.6%) and MOBA players (n=40, 34.5%). An additional group of healthy controls (n=37, 31.9%) with no gaming experience in the past 2 years was also included. High-resolution structural magnetic resonance imaging data were acquired using a 3-T scanner. Individualized differential structural covariance networks were constructed based on the cortical thickness values extracted from 68 brain regions using the Desikan-Killiany atlas. Statistical analysis included one-way ANOVA to identify significant structural covariance edges (SCEs), network-based statistic prediction analysis for weekly gaming hours, and support vector machine analysis for group classification.

**Results:**

One-way ANOVA identified 30 significant SCEs across the 3 groups (*P*<.001, false discovery rate corrected). Post hoc analysis (*P*<.02, Bonferroni corrected) revealed that, compared to the MOBA and control groups, the FPS group exhibited 2 dominant networks: a temporo-fronto-parietal network anchored in auditory regions and a visuo-sensorimotor network. Both gaming groups showed enhanced SCEs in visual-attentional networks compared to the control group. The network-based statistic–predict analysis demonstrated that structural covariance matrices could effectively predict weekly gaming hours in FPS players (*r*=0.34, 95% CI 0.26‐0.42). The positive edges primarily formed a temporo-fronto-parietal-occipital network, whereas the negative edges were centered on the entorhinal cortex. The support vector machine classifier successfully differentiated FPS players from controls (area under the curve=82.95%) and from MOBA players (area under the curve=72.37%).

**Conclusions:**

Long-term FPS and MOBA gaming experiences are associated with different brain structural network architectures. The uniqueness of FPS gaming lies in the extensive structural covariance between the primary auditory cortex and regions supporting visual attention and sensorimotor processing, which may reflect higher demands on cognitive skills. This suggests potential utility in auditory-visual rehabilitation and provides a theoretical basis for the assessment and selection of professional electronic sports players. However, the negative edges involving the entorhinal cortex in FPS players indicate that an overreliance on response learning strategies may come at the expense of the spatial memory system. Consequently, caution is warranted when applying such games to ameliorate age-related memory decline.

## Introduction

The relationship between video games and human cognitive plasticity has been a focal point of research over the past decades [1]. As electronic sports (esports) became an official event in the Olympic Games, video games, recognized as particularly captivating within the esports domain, gained popularity. Action video games (AVGs), a common subtype of video games [2], involve the rapid perception of high-load targets and the execution of actions under time pressure, as well as flexible switching between focused and divided attention to enhance attentional control [3]. These components of AVGs may enhance cognitive functions such as perception [4], attention [5,6], memory [7], and executive control [8], which are known to decline with aging and in neurodegenerative diseases [9,10]. Recent studies further suggest that video games can positively influence brain health and mental well-being [11,12]. Therefore, exploring the potential of AVGs to improve these cognitive functions could provide valuable insights for developing nonpharmacological intervention strategies aimed at mitigating age-related or neurodegenerative cognitive decline.

Traditional AVGs typically refer to first-person shooter (FPS) games. However, with the evolution of video games, certain video games that were not traditionally classified as AVGs now incorporate elements typically associated with AVGs. This includes the multiplayer online battle arena (MOBA) genre, which is sometimes referred to as action real-time strategy (RTS) games [1,3]. FPS games are characterized by players engaging in combat using firearms or other weapons within a first-person perspective environment, either individually or as part of a team. These games impose higher sensorimotor demands on players, requiring enhanced hand-eye coordination and rapid responses [13]. Players must quickly locate enemy positions, make swift decisions, aim accurately, and ultimately eliminate targets [14]. In MOBA games, players operate from a third-person perspective as part of a team. They must comprehend the characteristics of each character and collaboratively devise appropriate strategies to destroy the opposing team’s headquarters, which places higher demands on cognitive abilities, such as working memory and short-term memory [15].

Previous studies indicated that extensive experience with AVGs enhanced players’ performance on auditory tasks [16,17]. Specifically, for high-level FPS players, shorter auditory latency was associated with improved game performance [18]. Thus, acoustic information likely played an essential role for FPS players. Additionally, an electroencephalography study revealed that FPS players produced greater interactions than controls between auditory and visual networks and between auditory and attention networks, whereas MOBA players showed greater functional interactions than controls between visual and attention networks only [19]. Therefore, based on existing research [16-19], we hypothesized that the structural differences underlying auditory attention and other cognitive abilities may differ between long-term MOBA players and FPS players. To investigate this potential divergence in depth, selecting an appropriate neuroimaging technique is crucial. Although electroencephalography exhibits high temporal resolution, its spatial localization remains relatively limited [20]. While magnetoencephalography provides superior spatiotemporal resolution, its widespread application is constrained by prohibitive equipment and acquisition costs [21]. Furthermore, functional magnetic resonance imaging (MRI) data are prone to instability arising from factors such as head motion [22] and physiological noise [23]. In contrast, structural MRI, primarily used to acquire anatomical information of the brain, offers more stable image quality and relatively lower acquisition costs and is frequently used to assess alterations in structural brain connectivity [24]. Consequently, this study aimed to investigate, from an anatomical perspective, whether different types of video games exert distinct cognitive effects.

The phenomenon of structural covariance refers to the pattern in which structural differences in one brain region covary with differences in other regions, influenced by genetic, behavioral, and cognitive factors [25]. Initially, this approach was used to investigate cortical thickness (CT) across developmental stages and associated changes across multiple regions [26]. CT is defined as the distance between the pial surface and the white matter (WM), reflecting the thickness of the cortical gray matter (GM). In healthy adults, greater CT is generally associated with better cognitive abilities, such as auditory attention, visuospatial attention, and reward learning [27-30]. Studies have found that structural connectivity often mirrors patterns of functional connectivity [31], suggesting that regions exhibiting morphological covariance belong to the same functional networks [32,33], and this correspondence is related to human cognition [34], potentially continuing to be reshaped throughout the lifespan [35,36]. Therefore, compared to approaches that primarily reflect short-term functional connectivity between brain regions, structural covariance networks can reflect characteristics of brain connectivity over longer time scales [37].

Early structural covariance networks were constructed by calculating statistical correlations between regions based on morphological measurements across groups of subjects [38]. However, this approach lacked individual brain morphological information, limiting its application in studying individual variability in brain structure. A recent study has proposed a network template perturbation method for constructing a single structural covariance network using regional GM volume information [39]. This individualized structural covariance network analysis calculates the Pearson correlation coefficient between GM volumes across regions and constructs a reference structural covariance network from a group of healthy controls. A perturbed structural covariance network is then created by adding a patient to the control group. Therefore, the strength of this approach lies in its individual-level construction, which incorporates reference group information and explicitly reflects the degree of morphological variation in each patient’s brain regions relative to the reference group. Individualized differential structural covariance network (IDSCN) analysis has been successfully applied in multiple cross-sectional studies across different age groups and disease conditions, ranging from cognitive impairment in preterm children [40] and posttraumatic stress disorder in young-to-middle-aged adults [41] to Alzheimer disease in older adults [42]. Several longitudinal studies have demonstrated that long-term video game training induces changes in brain structure [43-46]. However, traditional methods, such as voxel-based morphometry and surface-based morphometry, primarily assess changes in individual brain regions and fail to capture correlational relationships among multiple regions. Therefore, the IDSCN approach, which combines similarity to functional connectivity with stability over extended time scales, may provide valuable insights into brain structural differences between FPS and MOBA players from a structural connectivity perspective. There are 2 commonly used measures in IDSCN, that is, GM volume and CT. As a product of surface area and CT, GM volume cannot distinguish the relative contributions of these variables [47].

Therefore, we chose CT as the morphological measure for constructing the IDSCN in this study. Unlike GM volume, CT does not require the inclusion of total intracranial volume as a covariate [48,49]. The NBS-Predict provides a distinct methodological advantage for identifying subnetworks of connectivity strength that are associated with behavioral measures [50]. We therefore applied the NBS-Predict to predict weekly gaming hours separately for the FPS and MOBA groups based on their respective structural covariance matrices. Furthermore, similar to a previous study [51], to assess the extent to which IDSCN analysis can differentiate between FPS and MOBA players, as well as between video game players and the control group, we used support vector machines (SVMs) to establish classification models for the comparisons between each pair of groups. Building on previous research [6,16,19], we hypothesized that significant differences in CT covariation exist among the FPS, MOBA, and control groups. Specifically, we anticipate that the auditory regions in the FPS group will exhibit stronger CT covariation with other brain regions compared to those in both the MOBA and control groups.

## Methods

### Inclusion and Exclusion Criteria

In accordance with a previous study [51], we defined gaming group players based on skill and time commitment. For gaming skill criteria, we referenced rankings published in November 2023 [52]. Inclusion criteria for the FPS group were (1) ≥5 years of experience in PC-based FPS, ≥5 hours per week of gameplay in the last 6 months, and ranked within the top 15% of all players; and (2) no MOBA experience or no MOBA gameplay within the last 2 years. Inclusion criteria for the MOBA group were (1) ≥5 years of experience in PC-based MOBA, ≥5 hours per week of gameplay in the last 6 months, and ranked within the top 15% of all players, and (2) no FPS experience or no FPS gameplay within the last 2 years. Inclusion criteria for the control group were no MOBA or FPS experience, no gameplay within the last 2 years, and no gaming experience in the last 6 months.

Exclusion criteria included visual or auditory impairments, metallic implants or electronic devices in the head, neurological or psychological disorders, alcohol or drug abuse, smoking, and claustrophobia.

### Participant Characteristics

Consistent with previous research [53], it was challenging to recruit a sufficient number of high-level female players; therefore, all participants in this study were men. All participants were right-handed undergraduates or postgraduates.

### Sampling Procedures

Participants were recruited through online advertisements posted at Shanghai University of Sport. As part of the initial screening process, potential candidates completed a comprehensive questionnaire designed to verify the predefined inclusion and exclusion criteria. This questionnaire also gathered genre-specific information from the MOBA and FPS groups, including their competitive rankings and average weekly gaming hours over the previous 6 months. The next step involved collecting high-resolution brain structural images on participants who met the experimental criteria.

### Sample Size, Power, and Precision

Sample size determination was informed by prior cross-sectional neuroimaging studies investigating video game genres [54,55]. To ensure adequate statistical power and robust effect sizes, we established a minimum target of 35 participants per group. A total of 116 participants were successfully recruited, including 39 (33.6%) FPS players, 40 (34.5%) MOBA players, and 37 (31.9%) healthy controls.

### Measures and Covariates

The primary measures in this study were individualized structural covariance edges (SCEs) constructed based on CT across 68 brain regions. Potential covariates, including age and years of education, were prespecified for inclusion in the statistical models if significant between-group differences were observed during demographic comparisons. Additionally, weekly gaming hours were collected as a behavioral measure for the predictive analysis.

### Data Collection and Instrumentation

Structural images were acquired using a 3-T Siemens Prisma MRI scanner with a 64-channel phased-array head coil and using a magnetization-prepared rapid gradient echo sequence (repetition time=2300 ms, echo time=2.98 ms, inversion time=450 ms, field of view=256 mm, flip angle=9°, voxel size=1×1×1 mm³, and 176 contiguous slices). The duration of the structural acquisition scan was 5 minutes.

### Calculation of CT

High-resolution brain structural images were converted from DICOM to NIFTI format using MRIconvert (version 2.1.0; Lewis Center for Neuroimaging), followed by a quality check of each participant’s anatomical images to confirm the absence of significant artifacts. Anatomical images for each participant were realigned to the anterior commissure–posterior commissure plane using the Statistical Parametric Mapping (SPM12) software package [56] within the MATLAB 2020b environment [57], ensuring a consistent origin at the anterior commissure [58]. Subsequently, the Computational Anatomy Toolbox (CAT12.8.1 [59]) based on SPM12 was used to preprocess the MRI anatomical images of the 3 groups and to compute the CT. The specific steps included (1) standardization was conducted using Diffeomorphic Anatomical Registration using Exponentiated Lie algebra [60], followed by segmentation of the images into GM, WM, and cerebrospinal fluid; (2) calculation of CT: CT was calculated as the distance between the inner and outer surfaces of the GM [61]; (3) images were smoothed with a 15 mm FWHM Gaussian kernel; and (4) each hemisphere was segmented into 34 cortical regions using the Desikan-Killiany atlas [62], and the CT for all 68 brain regions across participants was extracted to construct the IDSCN.

### Data Diagnostics

The mean CT of all participants was within 3 SDs of the group mean. All participants met the image quality criterion of grade C or above, and therefore, no data were excluded from further analysis.

### Construction of IDSCN

The construction process of the IDSCN was as follows [39]:

1. Construction of the reference structural covariance network (rSCN): All participants in the control group were used to construct the rSCN. Each edge in the network was derived from the Pearson correlation coefficient (r) calculated for the CT between each pair of brain regions. Thus, the rSCN can be represented as r_n_.
2. Construction of the perturbed network: By adding gamer k into the control group, a new structural covariance network (SCN) is constructed using n+1 participants, which can be represented as the perturbed network r_n+1_.
3. Construction of the individualized SCN: The difference between the perturbed network and the reference network is calculated as ∆r_n_ = r_n+1_−r_n_. In this manner, an individualized SCN is generated for each participant in the FPS and MOBA groups.
4. The weight of each edge in the IDSCN was calculated: Using the Desikan-Killiany atlas, a total of 4624 edges were obtained across 68 brain regions. The weight of each edge can be calculated as follows (equation 1):

(1) $$Z=\frac{∆SCN}{\left(1-rSCN^{2})/(n-1\right)}$$

The edges in the IDSCN represent how the inclusion of gamer k altered the covariance of pairs of brain regions in CT compared to the reference group.

### SCEs and Statistical Analysis

We used the Gretna toolbox [63] to conduct a one-way ANOVA based on the z scores of 4624 edges across the 3 groups. Multiple comparisons were controlled by applying a false discovery rate correction using the mafdr function, which implements the Benjamini-Hochberg procedure, at a threshold of *P*<.001 [64]. Significant edge differences for all participants were extracted, and post hoc comparisons were performed using Bonferroni correction in IBM SPSS Statistics (version 26.0; *P*<.05/3=.02) [65]. The results were visualized using BrainNet Viewer [66]. To further enhance the robustness of the results, we conducted an additional nonparametric permutation test (10,000 permutations) using network-based statistics (NBS) on the 4624 edges among the 3 groups [67], with the α level set at .05 and the *F* threshold corresponding to the minimum significant *F* value derived from the parametric tests. The demographic information was tested for normality using the Shapiro-Wilk test in SPSS (version 26.0). Age and years of education were analyzed using the Kruskal-Wallis H test, while weekly gaming hours were assessed using the Mann-Whitney *U* test for independent samples. The distribution of gaming levels between the FPS group and the MOBA group was analyzed using the 2-sample Wilcoxon rank-sum test.

### NBS-Predict

The analysis predicting weekly gaming hours based on structural covariance matrices was conducted in accordance with the official NBS-Predict guidelines [50]. The significance threshold for the suprathreshold edge selection algorithm was set at *P*<.01, with 10,000 permutations used for statistical testing in correlation mode. Input features were scaled using MinMaxScaler, hyperparameter optimization was conducted via a grid search algorithm, and the number of optimization steps was set to 5.

### Support Vector Machine Analysis

In machine learning, SVM represents a class of supervised learning models and serves as a classic binary classification tool [68]. The construction of the SVM is consistent with a previous study [51]. We used SVM to assess the stability of connectivity differences between groups. The SVM analysis was conducted using the fitsvm function in MATLAB 2023a [57]. All differential edges in the FPS and MOBA groups were considered as feature values, with mutual information used as the feature selection algorithm [69], ranking feature values based on their importance. Leave-one-out cross-validation is a special case of k-fold cross-validation, where the number of folds equals the number of participants. It is particularly suitable for use when the dataset is small, and there is no need to randomly split the dataset into training and testing sets [70]. Therefore, we used a linear kernel SVM classifier and used the leave-one-out cross-validation strategy to evaluate its performance. The evaluation metrics included area under the curve (AUC), accuracy, sensitivity, and specificity [71]. Furthermore, SVM analysis was conducted on all differential edges between the FPS and the control groups, as well as between the MOBA and control groups. The visualization of the SVM results was performed using GraphPad Prism 9.5 [72].

### Ethical Considerations

This study involving human participants was reviewed and approved by the Ethics Committee of Shanghai University of Sport (approval 102772024RT028). All procedures were conducted in accordance with the Declaration of Helsinki, as well as relevant local legislation and institutional requirements. Written informed consent was obtained from all participants prior to their involvement, explicitly including provisions that allow for the secondary analysis of the collected research data without the requirement for further consent. Participants were informed that their participation was voluntary and their right to withdraw from the study at any time without penalty or the need to provide a reason. To ensure privacy and confidentiality, all data were anonymized. Furthermore, we confirm that no individual participant can be identified in any figures, tables, or supplementary materials presented in this manuscript. Upon completion of the experimental procedures, each participant received compensation of Chinese Yuan 35 (US $5) for their time and participation.

## Results

### Descriptive Data

This study included 116 male participants, divided into 3 groups, aged 18 to 26 (mean 21.2, SD 1.9) years. The demographic and game information of the 3 groups is presented in Table 1. In the FPS group, 90% (35/39) of the players played *Counter-Strike 2*, and 26% (10/39) played *Apex Legends*, with 15% (6/39) playing both games. The MOBA group included 98% (39/40) *League of Legends* players and 2% (1/40) *Dota 2* players. No significant differences were found in age or years of education among the study groups. We also observed no significant differences in the weekly gaming hours or rank distribution between MOBA and FPS groups.

**Table 1.** Demographic and game information.

Item	FPS^a^ (n=39)	MOBA^b^ (n=40)	Control (n=37)	*P* value
Age (y), median (IQR)	21 (19.5‐21)^c^	21 (20‐23)	22 (19‐23)	.18^d^
Education (y), median (IQR)	14 (13‐14)	14 (13‐16)	13 (12‐16)	.15^d^
Weekly gaming hours, median (IQR)	21 (11‐34)	18 (10‐25)	N/A^e^	.18^f^
Rank (top percentage of all players), % (n/N)				
<1%	41 (16/39)^g^	52.5 (21/40)	N/A	.08^h^
1%‐5%	25.6 (10/39)	32.5 (13/40)	N/A	N/A
5%‐10%	17.9 (7/39)	7.5 (3/40)	N/A	N/A
10%‐15%	15.4 (6/39)	7.5 (3/40)	N/A	N/A

a FPS: first-person shooter.

b MOBA: multiplayer online battle arena.

c Nonnormally distributed data were presented as median (IQR).

d *P* value was obtained by the Kruskal-Wallis H test.

e N/A: not applicable.

f *P *value was obtained by the Mann-Whitney *U* test.

g The number of players at different levels was expressed as a percentage.

h *P* value was obtained by the Wilcoxon rank-sum test.

### SCE Results

**Figure 1.**
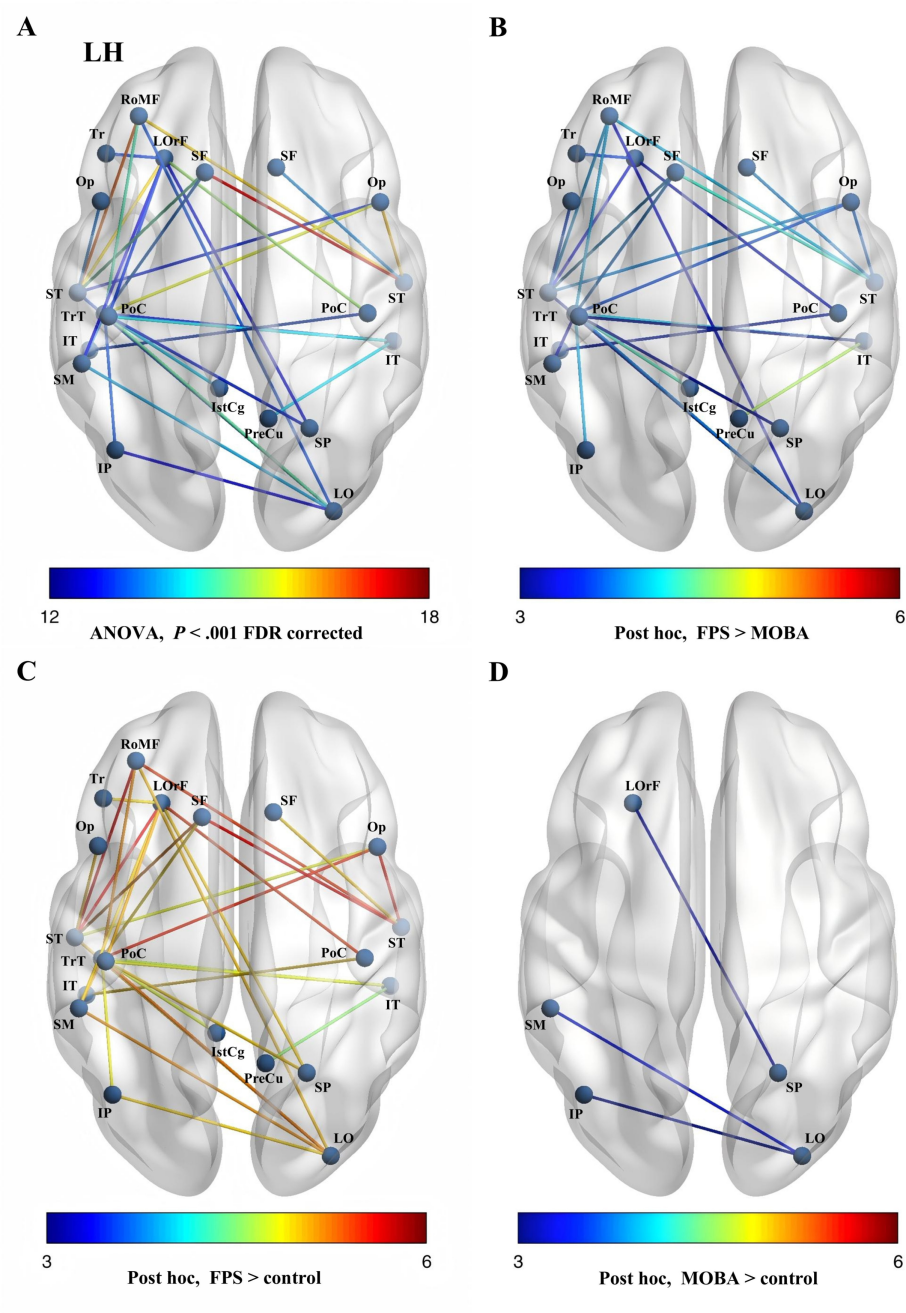
(A) Thirty edges that survived multiple comparison correction (*P*<.001, false discovery rate [FDR] corrected) in the one-way ANOVA across the 3 groups, with the color bar indicating *F* values. (B-D) Differences in edges observed between every 2 groups using the Bonferroni correction for multiple comparisons (*P*<.05/3=.02), where the color bar denotes *t* values. FPS: first-person shooter; IP: inferior parietal; IstCg: isthmuscingulate; IT: inferior temporal; LH: left hemisphere; LO: lateral occipital; LOrF: lateral orbitofrontal; MOBA: multiplayer online battle arena; PreCu: precuneus; Op: pars opercularis; PoC: postcentral; RoMF: rostral middle frontal; SF: superior frontal; SM: supramarginal; SP: superior parietal; ST: superior temporal; Tr: pars triangularis; TrT: transverse temporal.

The results of the one-way ANOVA for SCEs among the 3 groups are presented in Figure 1A and Table 2. A total of 30 SCEs were observed across the whole brain at the significant threshold of *P*<.001 (false discovery rate corrected). A consistent result was also obtained using an NBS analysis with 10,000 permutations (Multimedia Appendix 1).

**Table 2.** One-way ANOVA results for the 30 significant structural covariance edges (SCEs) among the 3 groups.

SCE	One-way ANOVA analysis		
	*F* test (*df*)	*P* value^a^	η^2^ (95% CI)
L^b^ ST^c^ - L LOrF^d^	15.86 (2, 113)	<.001	0.22 (0.09‐0.35)
L ST - L Op^e^	13.41 (2, 113)	<.001	0.19 (0.07‐0.32)
L ST-R^f^ Op	12.85 (2, 113)	<.001	0.19 (0.06‐0.31)
L ST - L RoMF^g^	16.71 (2, 113)	<.001	0.23 (0.10‐0.36)
L ST - L SF^h^	14.69 (2, 113)	<.001	0.21 (0.08‐0.33)
R ST - R Op	16.06 (2, 113)	<.001	0.22 (0.09‐0.35)
R ST - L RoMF	16.01 (2, 113)	<.001	0.22 (0.09‐0.35)
R ST - L SF	17.47 (2, 113)	<.001	0.24 (0.11‐0.37)
R ST - R SF	13.53 (2, 113)	<.001	0.19 (0.07‐0.32)
L TrT^i^ - R Op	15.61 (2, 113)	<.001	0.22 (0.09‐0.34)
L TrT - L RoMF	14.54 (2, 113)	<.001	0.20 (0.08‐0.33)
L TrT - L SF	13.30 (2, 113)	<.001	0.19 (0.07‐0.31)
L TrT - L IP^j^	13.15 (2, 113)	<.001	0.19 (0.06‐0.31)
L TrT - L SM^k^	13.47 (2, 113)	<.001	0.19 (0.07‐0.32)
L TrT - R SP^l^	12.84 (2, 113)	<.001	0.18 (0.06‐0.31)
L TrT - R LO^m^	14.81 (2, 113)	<.001	0.21 (0.08‐0.33)
L TrT - R IT^n^	12.73 (2, 113)	<.001	0.18 (0.06‐0.31)
L ST - R LO	13.02 (2, 113)	<.001	0.19 (0.06‐0.31)
L TrT - L IstCg^o^	13.88 (2, 113)	<.001	0.20 (0.07‐0.32)
R IT - R PreCu^p^	13.97 (2, 113)	<.001	0.20 (0.07‐0.32)
R IT - L PoC^q^	14.22 (2, 113)	<.001	0.20 (0.08‐0.33)
L IT - R PoC	13.05 (2, 113)	<.001	0.18 (0.06‐0.31)
R LO - L RoMF	13.16 (2, 113)	<.001	0.19 (0.06‐0.31)
L LOrF - L Tr^r^	13.01 (2, 113)	<.001	0.18 (0.06‐0.31)
L LOrF - R PoC	15.14 (2, 113)	<.001	0.21 (0.08‐0.34)
R LO - L IP	12.54 (2, 113)	<.001	0.18 (0.06‐0.30)
R LO - L SM	13.69 (2, 113)	<.001	0.20 (0.07‐0.32)
L LOrF - R SP	12.74 (2, 113)	<.001	0.18 (0.06‐0.31)
L LOrF - L SM	12.56 (2, 113)	<.001	0.18 (0.06‐0.30)
L LOrF - L PoC	12.94 (2, 113)	<.001	0.19 (0.06‐0.31)

a *P* value was adjusted using false discovery rate correction.

b L: left hemisphere.

c ST: superior temporal.

d LOrF: lateral orbitofrontal.

e Op: pars opercularis.

f R: right hemisphere.

g RoMF: rostral middle frontal.

h SF: superior frontal.

i TrT: transverse temporal.

j IP: inferior parietal.

k SM: supramarginal.

l SP: superior parietal.

m LO: lateral occipital.

n IT: inferior temporal.

o IstCg: isthmuscingulate.

p PreCu: precuneus.

q PoC: postcentral.

r Tr: pars triangularis

Post hoc analysis indicated that compared to the MOBA and control groups, the FPS group displayed 2 dominant networks. These consisted of a temporo-fronto-parietal network anchored in auditory regions, specifically the superior and transverse temporal gyri, and a visuo-sensorimotor network involving the inferior temporal gyrus and postcentral gyrus (Figure 1B and C and Table 3).

Additionally, both gaming groups exhibited significantly enhanced connectivity within visual-attentional networks relative to the control group (Figures 1C and 1D and Table 3).

All the significant SCEs mentioned earlier were treated as feature values using the MI algorithm for importance ranking, with lower values for greater significance of the feature in distinguishing between each pair of groups. Detailed results are presented in Multimedia Appendix 2.

**Table 3.** Post hoc results for the 30 significant structural covariance edges (SCEs) among the 3 groups.

SCEs	Post hoc analysis								
	FPS^a^>MOBA^b^			FPS>control			MOBA>control		
	*t* test (*df*)	*P* value	Cohen *d* (95% CI)^c^	*t* test (*df*)	*P* value	Cohen *d* (95% CI)	*t* test (*df*)	*P* value	Cohen *d* (95% CI)
L^d^ ST^e^ - L LOrF^f^	3.29 (76)	.004	0.74 (0.28‐1.20)	5.60 (73)	<.001	1.28 (0.78‐1.79)	2.39 (74)	.06	N/A^g^
L ST - L Op^h^	3.57 (76)	.002	0.80 (0.34‐1.27)	5.03 (73)	<.001	1.15 (0.66‐1.65)	1.53 (74)	.38	N/A
L ST - R^i^ Op	3.77 (76)	.001	0.85 (0.38‐1.32)	4.81 (73)	<.001	1.10 (0.61‐1.60)	1.12 (74)	.79	N/A
L ST - L RoMF^j^	3.82 (76)	.001	0.86 (0.39‐1.33)	5.66 (73)	<.001	1.30 (0.80‐1.80)	1.93 (74)	.17	N/A
L ST - L SF^k^	3.81 (76)	.001	0.86 (0.39‐1.32)	5.23 (73)	<.001	1.20 (0.70‐1.70)	1.51 (74)	.40	N/A
R ST - R Op	3.69 (76)	.001	0.83 (0.36‐1.30)	5.57 (73)	<.001	1.28 (0.78‐1.78)	1.96 (74)	.16	N/A
R ST - L RoMF	3.92 (76)	<.001	0.88 (0.41‐1.35)	5.49 (73)	<.001	1.26 (0.76‐1.76)	1.65 (74)	.30	N/A
R ST - L SF	4.24 (76)	<.001	0.95 (0.48‐1.43)	5.68 (73)	<.001	1.30 (0.80‐1.81)	1.53 (74)	.39	N/A
R ST - R SF	3.77 (76)	.001	0.85 (0.38‐1.32)	4.98 (73)	<.001	1.14 (0.65‐1.64)	1.29 (74)	.60	N/A
L TrT^l^ - R Op	3.55 (76)	.002	0.80 (0.33‐1.26)	5.51 (73)	<.001	1.26 (0.76‐1.76)	2.04 (74)	.13	N/A
L TrT - L RoMF	3.87 (76)	.001	0.87 (0.40‐1.34)	5.18 (73)	<.001	1.19 (0.70‐1.68)	1.40 (74)	.49	N/A
L TrT - L SF	3.70 (76)	.001	0.83 (0.36‐1.30)	4.96 (73)	<.001	1.14 (0.65‐1.63)	1.34 (74)	.54	N/A
L TrT - L IP^m^	3.88 (76)	.001	0.87 (0.40‐1.34)	4.84 (73)	<.001	1.11 (0.62‐1.60)	1.04 (74)	.90	N/A
L TrT - L SM^n^	3.03 (76)	.009	0.68 (0.22‐1.14)	5.16 (73)	<.001	1.18 (0.69‐1.68)	2.20 (74)	.09	N/A
L TrT - R SP	3.10 (76)	.007	0.70 (0.24‐1.16)	5.02 (73)	<.001	1.15 (0.66‐1.64)	1.99 (74)	.15	N/A
L TrT - R LO^o^	3.73 (76)	.001	0.84 (0.37‐1.31)	5.29 (73)	<.001	1.21 (0.72‐1.71)	1.64 (74)	.31	N/A
L TrT - R IT^p^	4.02 (76)	<.001	0.90 (0.43‐1.37)	4.65 (73)	<.001	1.07 (0.58‐1.56)	0.71 (74)	>.99	N/A
L ST - R LO	3.10 (76)	.007	0.70 (0.24‐1.16)	5.06 (73)	<.001	1.16 (0.67‐1.66)	2.03 (74)	.13	N/A
L TrT - L IstCg^q^	4.30 (76)	<.001	0.97 (0.49‐1.44)	4.78 (73)	<.001	1.10 (0.61‐1.59)	0.57 (74)	>.99	N/A
R IT - R PreCu^r^	4.62 (76)	<.001	1.04 (0.56‐1.52)	4.52 (73)	<.001	1.04 (0.55‐1.52)	-0.02 (74)	>.99	N/A
R IT - L PoC^s^	4.41 (76)	<.001	0.99 (0.52‐1.47)	4.79 (73)	<.001	1.10 (0.61‐1.59)	0.47 (74)	>.99	N/A
L IT - R PoC	3.38 (76)	.003	0.76 (0.30‐1.22)	5.00 (73)	<.001	1.15 (0.65‐1.64)	1.70 (74)	.28	N/A
R LO - L RoMF	3.27 (76)	.004	0.74 (0.27‐1.20)	5.05 (73)	<.001	1.16 (0.66‐1.65)	1.86 (74)	.20	N/A
L LOrF - L Tr^t^	3.53 (76)	.002	0.79 (0.33‐1.26)	4.95 (73)	<.001	1.14 (0.64‐1.63)	1.49 (74)	.41	N/A
L LOrF - R PoC	3.30 (76)	.004	0.74 (0.30‐1.20)	5.46 (73)	<.001	1.25 (0.75‐1.75)	2.24 (74)	.08	N/A
R LO - L IP	2.06 (76)	.12	N/A	4.98 (73)	<.001	1.14 (0.65‐1.64)	2.98 (74)	.01	0.68 (0.21‐1.15)
R LO - L SM	1.90 (76)	.18	N/A	5.18 (73)	<.001	1.19 (0.69‐1.68)	3.33 (74)	.003	0.76 (0.29‐1.23)
L LOrF - R SP	1.94 (76)	.16	N/A	5.01 (73)	<.001	1.15 (0.66‐1.64)	3.12 (74)	.007	0.71 (0.24‐1.18)
L LOrF - L SM^u^	2.66 (76)	.03	N/A	5.01 (73)	<.001	1.15 (0.66‐1.64)	2.41 (74)	.05	N/A
L LOrF - L PoC	2.52 (76)	.04	N/A	5.09 (73)	<.001	1.17 (0.67‐1.66)	2.63 (74)	.03	N/A

a FPS: first-person shooter.

b MOBA: multiplayer online battle arena.

c Cohen *d* (95% CI) is only reported if the level of significance is <.02.

d L: left hemisphere.

e ST: superior temporal.

f LOrF: lateral orbitofrontal.

g N/A: not applicable.

h Op: pars opercularis.

i R: right hemisphere.

j RoMF: rostral middle frontal.

k SF: superior frontal.

l TrT: transverse temporal.

m IP: inferior parietal.

n SM: supramarginal.

o LO: lateral occipital.

p IT: inferior temporal.

q IstCg: isthmuscingulate.

r PreCu: precuneus.

s PoC: postcentral.

t Tr: pars triangularis.

u SM: supramarginal.

### NBS-Predict Results

**Figure 2.**
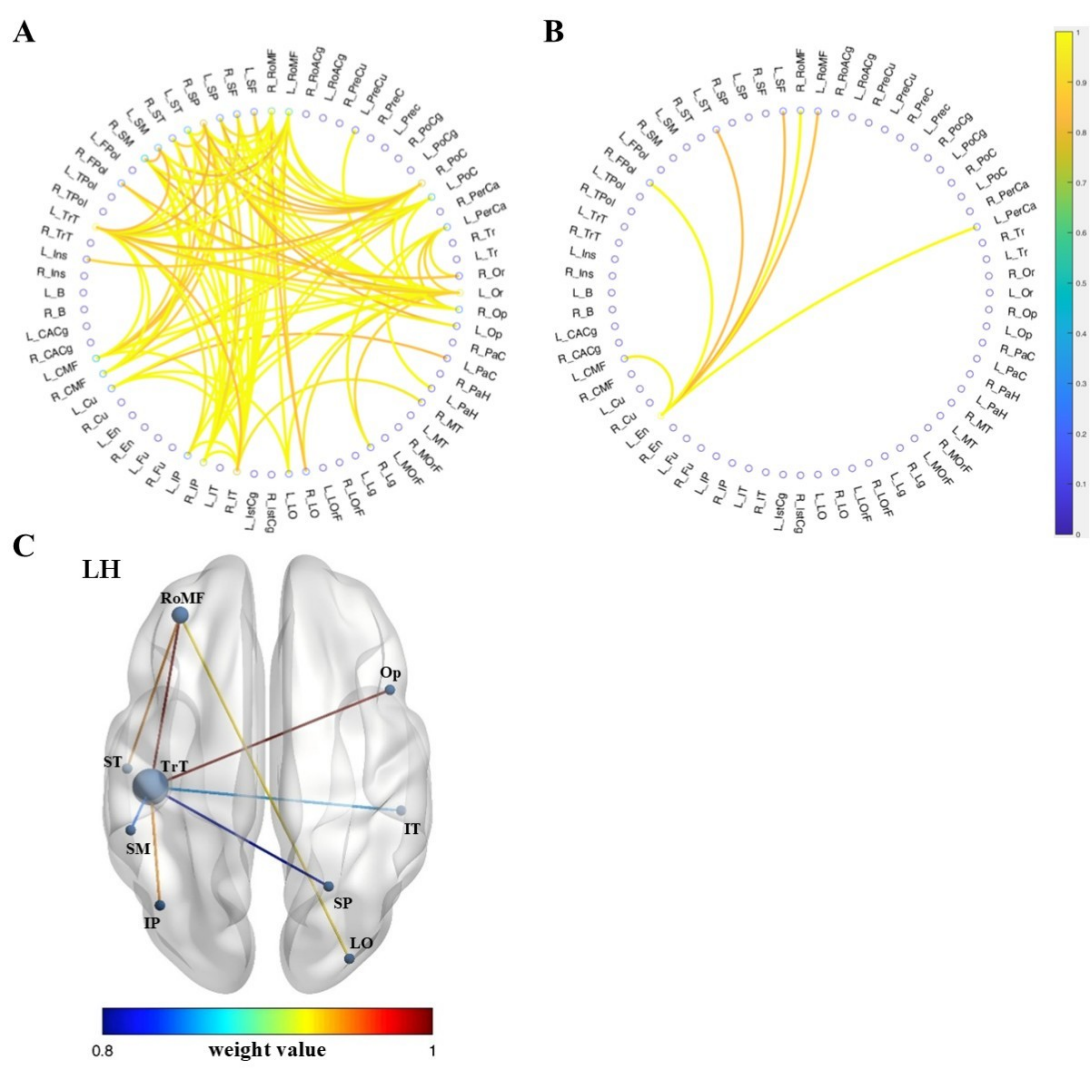
Using linear regression within the NBS-Predict framework, weekly gaming hours in the first-person shooter group (FPS) were predicted from the structural covariance matrix, showing a Pearson correlation of 0.34 (95% CI 0.26‐0.42) between predicted and actual weekly gaming hours. Panel A shows 100 positive edges under a weight threshold of 0.8. Panel B illustrates the 7 negative edges under the same threshold. Panel C presents the structural covariance edges in which the positive edges overlap with those identified in Figure 1B (ie, the differential edges representing FPS>MOBA). Nodes and edges are depicted by size and color according to the nodal degree and weight, respectively. CMF: caudal middle frontal; Cu: cuneus; En: entorhinal; FPoL: frontal pole; Ins: insula; IP: inferior parietal; IstCg: isthmus cingulate; IT: inferior temporal; Lg: lingual; LH: left hemisphere; LO: lateral occipital; LOrF: lateral orbitofrontal; MOBA: multiplayer online battle arena; MT: middle temporal; Op: pars opercularis; Or: pars orbitalis; PaC: paracentral; PaH: parahippocampal; PerCa: pericalcarine; PoC: postcentral; PreCu: precuneus; RoMF: rostral middle frontal; SF: superior frontal; SM: supramarginal; SP: superior parietal; ST: superior temporal; Tr: pars triangularis; TrT: transverse temporal.

The weekly gaming time of the FPS group was predicted using the NBS-Predict based on individual structural covariance matrices. The predicted weekly gaming time showed a significant correlation with the actual values, with a Pearson correlation coefficient (*r*) of 0.34 (95% CI 0.26‐0.42). Within the linear regression model, we identified 100 positive edges and 7 negative edges (connecting 34 brain regions) with weight values >0.8 (Table 4 and Figure 2), where higher edge weights indicate greater contribution to the model. The positive edges constituted a connected network centered on the auditory-related transverse temporal gyrus and extending into frontal, parietal, and occipital lobes, involving regions such as the inferior temporal gyrus, superior temporal gyrus, pars orbitalis, rostral middle frontal gyrus, superior parietal gyrus, inferior parietal gyrus, and postcentral gyrus (Figure 2A). The negative edges primarily reflected connections between the entorhinal cortex and several frontal regions (eg, rostral middle frontal, caudal middle frontal, and frontal pole), the pericalcarine cortex, and the superior temporal cortex (Figure 2B). Furthermore, this pattern of positive edges showed substantial overlap with the differential network of the FPS group relative to the MOBA group (Figure 1B), with overlapping connections illustrated in Figure 2C. Additionally, a validation analysis was performed by increasing the weight threshold to 0.9, which revealed that the identified significant connections remained highly consistent with those obtained under the 0.8 threshold (Table 4 and Multimedia Appendix 3 and Multimedia Appendix 4).

No significant predictive effect was observed when using the structural covariance matrices of the MOBA group to predict their weekly gaming time.

**Table 4.** Nodes and nodal degrees of structural covariance edges correlated with weekly gaming hours in the first-person shooter group under the weight value >0.8 (*P* value <.01 and weight value >0.8).

Positive edges		Negative edges	
Node^a^	Degree	Node	Degree
L^b^ TrT^c^	17	L En^d^	7
L Or^e^	16	L CMF^f^	1
R IT^g^	13	L PerCa^h^	1
R PoC^i^	13	L RoMF^j^	1
R SP^k^	13	R RoMF	1
R IP^l^	12	L SF^m^	1
R RoMF	11	L ST^n^	1
L RoMF	10	R FPol^o^	1
L CMF	8	N/A^p^	N/A
R SM^q^	8	N/A	N/A
L PoC	7	N/A	N/A
L ST	7	N/A	N/A
R CMF	6	N/A	N/A
L SM	6	N/A	N/A
R Op^r^	5	N/A	N/A
L PerCa	5	N/A	N/A
L Cu^s^	4	N/A	N/A
L IP	4	N/A	N/A
R SF	4	N/A	N/A
L SP	4	N/A	N/A
R FPol	4	N/A	N/A
L LO^t^	3	N/A	N/A
R LO	3	N/A	N/A
L SF	3	N/A	N/A
R ST	3	N/A	N/A
R Lg^u^	2	N/A	N/A
L Op	2	N/A	N/A
R Or	2	N/A	N/A
R MT^v^	1	N/A	N/A
L PaH^w^	1	N/A	N/A
L PaC^x^	1	N/A	N/A
L PreCu^y^	1	N/A	N/A
L Ins^z^	1	N/A	N/A

a Node: Abbreviations correspond to cortical regions defined by the Desikan-Killiany atlas.

b L: left hemisphere.

c TrT: transverse temporal.

d En: entorhinal.

e Or: pars orbitalis.

f CMF: caudal middle frontal.

g IT: inferior temporal.

h PerCa: pericalcarine.

i PoC: postcentral.

j RoMF: rostral middle frontal.

k SP: superior parietal.

l IP: inferior parietal.

m SF: superior frontal.

n ST: superior temporal.

o FPol: frontal pole.

p N/A: not applicable.

q SM: supramarginal.

r Op: pars opercularis.

s Cu: cuneus.

t LO: lateral occipital.

u Lg: lingual.

v MT: middle temporal.

w PaH: parahippocampal.

x PaC: paracentral.

y PreCu: precuneus.

z Ins: insula.

### SVM Results on Testing Set

**Figure 3.**
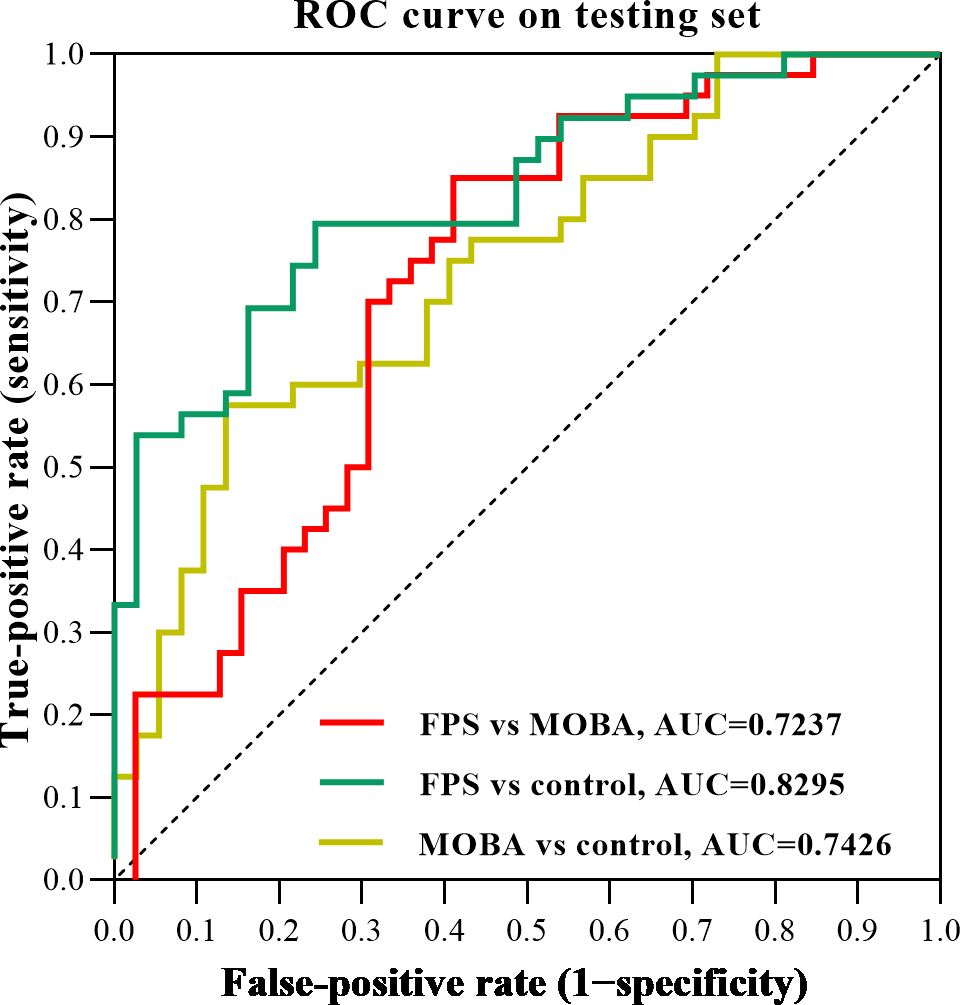
The receiver operating characteristic (ROC) curve for the comparisons between each 2 groups within the support vector machine classifier. AUC: area under the curve; FPS: first-person shooter; MOBA: multiplayer online battle arena.

The classification results showed that the FPS group could be differentiated from the MOBA group based on high AUC (72.37%), accuracy (70.89%), sensitivity (85%), and specificity (56.41%). The AUC, accuracy, sensitivity, and specificity were 82.95%, 73.68%, 64.10%, and 83.78%, respectively, for the classification between the FPS and control groups. The AUC, accuracy, sensitivity, and specificity were 74.26%, 67.53%, 57.50%, and 78.38%, respectively, for the classification between the MOBA and control groups (Figure 3).

## Discussion

### Principal Findings

This study aimed to examine whether long-term FPS and MOBA gaming experience is associated with the covariation of CT across brain regions, thereby elucidating the potential corresponding cognitive functions. Our results corroborated the initial hypotheses, revealing that compared to the MOBA and control groups, FPS players exhibited differences in CT covariation between the auditory cortex and the frontal, occipital, and parietal regions. These networks, involved in auditory processing, visual perception, attentional control, cognitive flexibility, and sensorimotor integration, also demonstrated predictive value for weekly gaming hours. Compared to the control group, MOBA players showed differences in CT covariation between the lateral occipital cortex and the inferior parietal and supramarginal regions, as well as between the superior parietal and the lateral orbitofrontal cortex. Furthermore, significant connectivity edges differentiating the FPS and MOBA groups were used as input features for an SVM classifier, and the results indicated that these structural signatures effectively discriminated between the 2 player groups.

### Comparison With Prior Work

Previous research has found that long-term experience with RTS games can enhance WM connectivity in the occipito-parietal regions [8], and such structural changes may further drive functional reorganization in frontoparietal and parieto-occipital brain regions [73]. Classical video game genres primarily include FPS, MOBA, and RTS. Early studies have demonstrated the benefits of video games for visual attention [5,74]. In this study, we observed covariation within frontal-parietal-occipital regions among MOBA and FPS players, which may reflect the influence of shared cognitive demands associated with these game genres. Two review articles on the cognitive benefits of video game training have suggested that not all video games elicit the same cognitive effects [3,75]. Consistent with this perspective, our findings revealed that FPS players exhibited more extensive covariation patterns across auditory, visual, and sensorimotor networks. This may indicate that compared with MOBA players, whose covariation patterns were primarily confined to visual-attentional networks, FPS gameplay imposes more comprehensive cognitive demands on players.

Longitudinal intervention studies investigating video game training have reported increases in GM volume in the superior temporal cortex [45] and the dorsolateral prefrontal cortex [76]. The superior and transverse temporal gyri are key anatomical substrates for auditory processing [77,78], whereas prefrontal regions are primarily associated with cognitive flexibility [79]. These findings are consistent with the temporal-prefrontal covariance patterns observed in our study. This correspondence suggests that FPS players may rely on dynamic interactions between auditory processing networks and attentional control networks [19].

Visual stimuli, a core element of AVGs, produce stronger visual motion effects in first-person perspectives compared to third-person perspectives [80]. A cross-sectional study found that AVG experience might lead to improvements in attention observed in both the visual and auditory domains [17], and AVG training has been shown to improve phonemic decoding and visual attention in children with developmental dyslexia [81]. Long-term FPS intervention could increase GM volume in visual regions such as the middle occipital gyrus [45]. Notably, alterations in occipital networks have been linked to neurodegenerative conditions such as Lewy body dementia [10]. Moreover, auditory-perceptual game-based training has been shown to improve cognitive performance in older adults, an effect that depends on the integrity of occipito-temporal WM connections [82]. Thus, the observed auditory-visual covariance, which positively correlates with weekly gaming hours in FPS players, implies that FPS-like gaming paradigms may offer a promising approach to maintaining brain health and mitigating age-related cognitive decline.

Research has shown that long-term FPS interventions may increase CT of the sensorimotor regions [44]. The superior parietal and inferior parietal regions were associated with the execution control of visually related motor responses [83]. Moreover, the superior parietal cortex also serves as a critical region for hand movements [84]. In an auditory-motor integration task, the superior temporal and supramarginal regions were co-activated in pianists [85] who shared certain common features with AVG players in frequent hand movements. The joint engagement of superior temporal and inferior parietal cortices functions to modulate motor responses to auditory stimuli in relation to visual stimuli [86]. FPS games have been examined in motor skill research due to their distinct kinematic characteristics [13,14]. Furthermore, these games are rich in auditory cues. For example, players often rely on enemy footsteps to localize opponents and execute rapid sequences of actions to eliminate them. The observed SCEs between auditory and sensorimotor networks may imply an association with integrated audio-motor processing.

We found that the negative SCEs between the entorhinal cortex and the frontal regions as well as the superior temporal gyrus in FPS players could effectively predict weekly gaming hours. This is a noteworthy phenomenon that may be linked to negative cognitive effects associated with FPS gaming. West et al [43] found that spatial navigation strategies can increase GM volume in the entorhinal cortex. In contrast, the use of in-game GPS guidance in FPS games—directing players to subsequent locations or events—may encourage greater reliance on response learning strategies, thereby indirectly contributing to reductions in hippocampal and entorhinal regions involved in spatial navigation. Additionally, the observed negative edge between the entorhinal cortex and superior temporal gyrus resembles the reduced hippocampal-superior temporal functional connectivity reported by Benady-Chorney et al [87] in AVG players, which may further indicate potential memory impairment. With advancing age, humans tend to shift toward response learning strategies [88], and FPS gaming may exacerbate such aging-related cognitive risks. Therefore, future game design should aim to balance elements related to response learning and spatial navigation strategies to safeguard the memory system.

### Limitations

This study has several limitations. First, the cross-sectional design precludes causal conclusions about the relationship between game type and brain structure. It is also possible that inherent brain structure specificity leads players to perform better in FPS games rather than MOBA games. Second, although we observed covariation of CT between the auditory cortex and several other regions in FPS players, no auditory tasks were designed and conducted, which limits our ability to directly correlate auditory behavior with structural network changes. Future research should use a longitudinal intervention design, coupled with task-based functional MRI, to explore the effects of FPS and MOBA games on players’ auditory attention. Finally, while IDSCN has been shown to resemble functional networks in several studies [31-33] and offers substantial advantages by overcoming the limitations of group-level structural covariance analysis through individual-level assessments, it serves only as an indirect marker of neuroplasticity. Its biological basis remains incompletely understood, and interpretation should be approached with caution.

### Conclusions

This study characterizes the distinct structural network architectures associated with long-term FPS and MOBA gaming. The findings indicate that while both genres engage visual-attentional networks, FPS gaming is uniquely associated with extensive structural covariance between the primary auditory cortex and regions governing visual attention, sensorimotor processing, and multisensory integration. These distinct patterns likely reflect the broader cognitive demands of FPS gaming, supporting the potential utility of FPS-based paradigms in auditory and visual rehabilitation [89-92]. Additionally, the identified structural features offer a theoretical foundation for the assessment and selection of professional esports players. However, the observed negative edges involving the entorhinal cortex warrant caution. This may indicate that the heavy reliance on response learning strategies in FPS gaming comes at the expense of spatial navigation systems dependent on the entorhinal cortex. This potential trade-off is particularly relevant for designing game-based interventions aimed at mitigating age-related cognitive decline. Given the cross-sectional nature of this study, these structural associations cannot confirm causality. Future research should prioritize longitudinal designs combined with auditory behavioral tasks to verify whether these gaming habits actively induce neuroplasticity or reflect preexisting traits and to further elucidate the balance between cognitive benefits and potential risks to the spatial memory system.

## Supplementary material

10.2196/79976 Multimedia Appendix 1 A nonparametric test was conducted using network-based statistics (NBS) with 10,000 permutations on 4624 SCEs among the 3 groups, at an α level of .05 and with an *F *value threshold ≥12.54. The 30 significant SCEs identified (*P*<.001) were consistent with the results obtained from the parametric test.

10.2196/79976 Multimedia Appendix 2 Importance ranking of significant structural covariance edges based on the MI algorithm in support vector machine analysis.

10.2196/79976 Multimedia Appendix 3 Nodes and nodal degrees of structural covariance edges correlated with weekly gaming hours in the first-person shooter group under the weight value >0.9.

10.2196/79976 Multimedia Appendix 4 Using linear regression within the network-based statistic–Predict framework, weekly gaming hours in the first-person shooter group were predicted from the structural covariance matrix, showing a Pearson correlation of 0.34 (95% CI 0.26-0.42) between predicted and actual weekly gaming hours. Panel A shows 79 positive edges under a weight threshold of 0.9. Panel B illustrates the 4 negative edges under the same threshold.

10.2196/79976 Multimedia Appendix 5 Individualized Differential Structural Covariance Network code1.

10.2196/79976 Multimedia Appendix 6 Individualized Differential Structural Covariance Network code2.

10.2196/79976 Multimedia Appendix 7 Support vector machine code.

## Abbreviations

AUC: area under the curve
AVG: action video game
CAT: Computational Anatomy Toolbox
CT: cortical thickness
esports: electronic sports
FPS: first-person shooter
Gen-AI: generative artificial intelligence
GM: gray matter
IDSCN: Individualized Differential Structural Covariance Network
MOBA: multiplayer online battle arena
MRI: magnetic resonance imaging
NBS: network-based statistic
rSCN: Reference structural covariance network
RTS: real-time strategy
SCE: structural covariance edge
SCN: structural covariance network
SPM: Statistical Parametric Mapping
SVM: support vector machine
WM: white matter
