# Supplementary figures and images for: Brain Structural Covariance Networks in Long-Term First-Person Shooter and Multiplayer Online Battle Arena Players: Cross-Sectional Study
Source: JMIR Serious Games. 2026 May 4;14:e79976. doi: 10.2196/79976 (PMC13150961; doi:10.2196/79976)

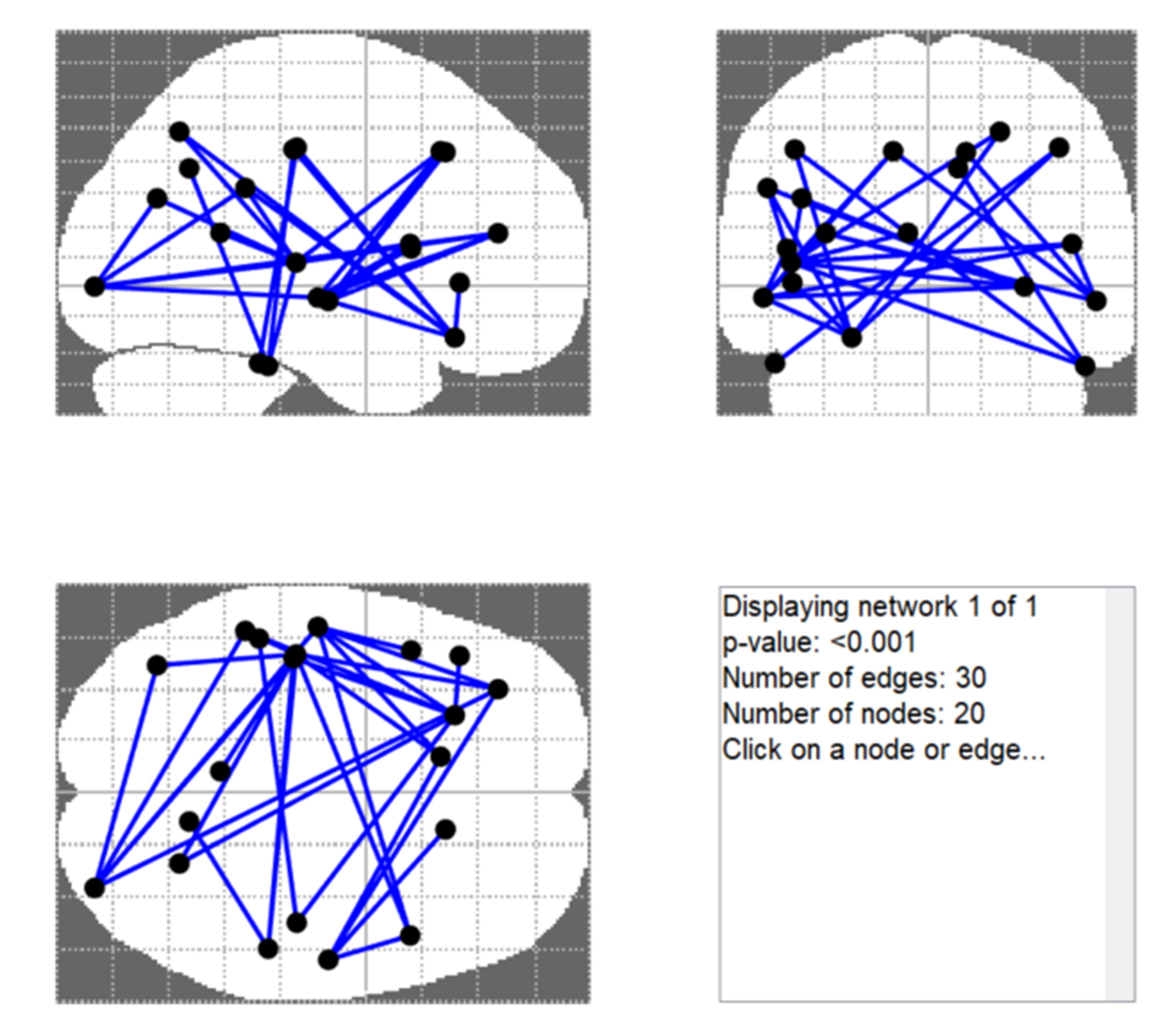

Supplement: Multimedia Appendix 1 [file games-v14-e79976-s001.png]

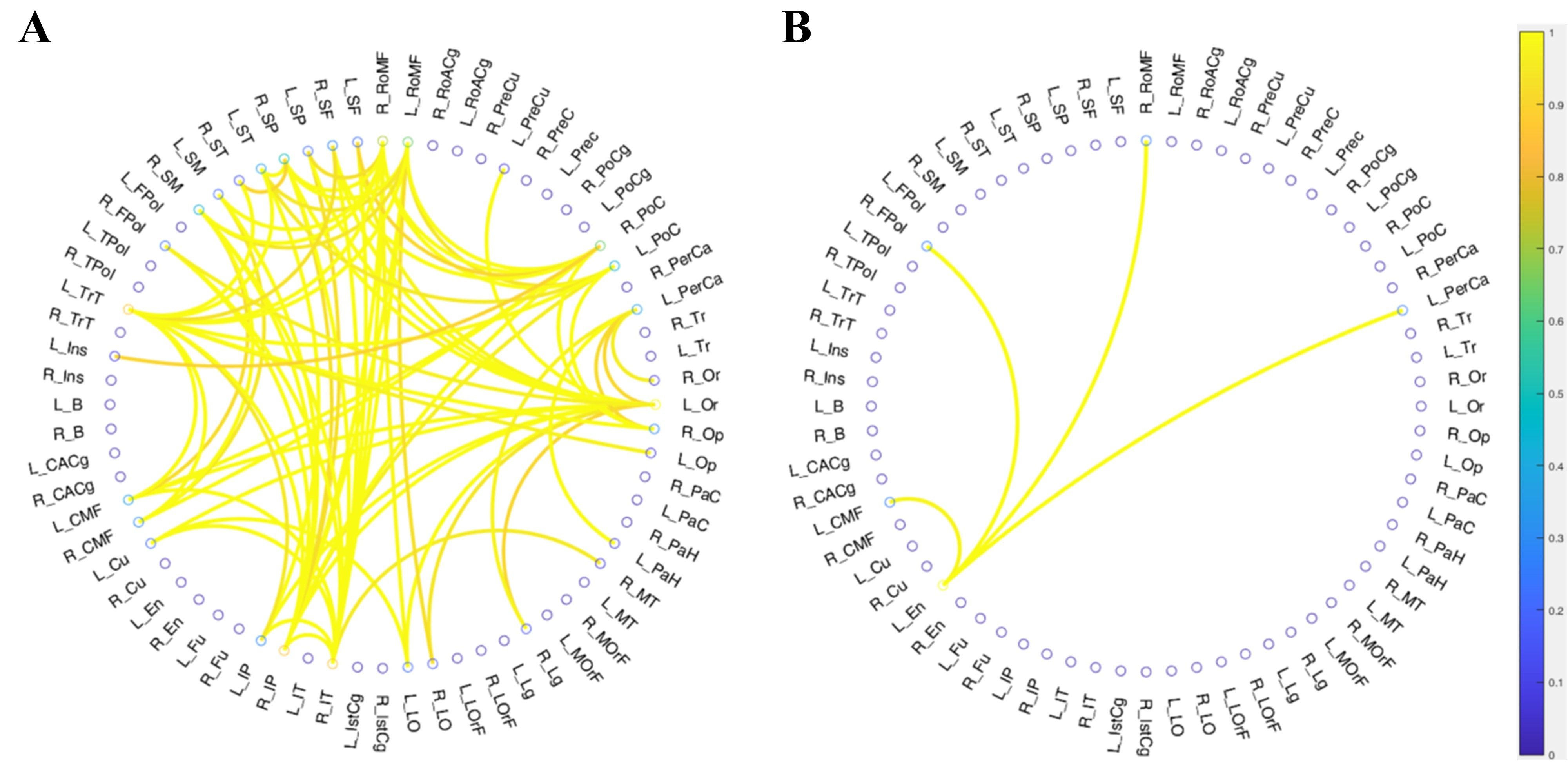

Supplement: Multimedia Appendix 4 [file games-v14-e79976-s004.png]
