# Supplementary material for: Brain Structural Covariance Networks in Long-Term First-Person Shooter and Multiplayer Online Battle Arena Players: Cross-Sectional Study
Source: JMIR Serious Games. 2026 May 4;14:e79976. doi: 10.2196/79976 (PMC13150961; doi:10.2196/79976)
Supplement: Multimedia Appendix 2 [file games-v14-e79976-s002.docx]

**SUPPLEMENTARY MATERIALS**

**Brain Structural Covariance Networks in Long-Term First Person Shooter and Multiplayer Online Battle Arena Players: A Cross-Sectional Study**

**Multimedia Appendix 2.** Importance ranking of significant SCEs based on the MI algorithm in SVM analysis.

| Structural covariance edges^a^ | Importance ranking^b^ | | |
| --- | --- | --- | --- |
|  | FPS>MOBA | FPS>Control | MOBA>Control |
| L ST - L LOrF | 12 | 5 | N/A^c^ |
| L ST - L Op | 13 | 14 | N/A |
| L ST - R Op | 17 | 26 | N/A |
| L ST - L RoMF | 10 | 13 | N/A |
| L ST - L SF | 9 | 24 | N/A |
| R ST - R Op | 2 | 4 | N/A |
| R ST - L RoMF | 7 | 9 | N/A |
| R ST - L SF | 16 | 1 | N/A |
| R ST - R SF | 25 | 22 | N/A |
| L TrT - R Op | 23 | 18 | N/A |
| L TrT - L RoMF | 5 | 23 | N/A |
| L TrT - L SF | 14 | 21 | N/A |
| L TrT - L IP | 4 | 6 | N/A |
| L TrT - L SM | 15 | 3 | N/A |
| L TrT - R SP | 21 | 12 | N/A |
| L TrT - R LO | 20 | 7 | N/A |
| L TrT - R IT | 19 | 28 | N/A |
| L ST - R LO | 6 | 2 | N/A |
| L TrT - L IstCg | 1 | 16 | N/A |
| R IT - R PreCu | 3 | 29 | N/A |
| R IT - L PoC | 11 | 30 | N/A |
| L IT - R PoC | 22 | 19 | N/A |
| R LO - L RoMF | 24 | 20 | N/A |
| L LOrF - L Tr | 8 | 15 | N/A |
| L LOrF - R PoC | 18 | 25 | N/A |
| R LO - L IP | N/A | 27 | 2 |
| R LO - L SM | N/A | 17 | 3 |
| L LOrF - R SP | N/A | 10 | 1 |
| L LOrF - L SM | N/A | 8 | N/A |
| L LOrF - L PoC | N/A | 11 | N/A |

^a^Structural covariance edges: Corresponding abbreviations based on the Desikan-Killiany atlas. IP, Inferior parietal; IT, Inferior temporal; IstCg, Isthmuscingulate; L, Left Hemisphere; LO, Lateral occipital; LOrF, Lateral orbitofrontal; Op, Pars opercularis; Tr, Pars triangularis; PoC, Postcentral; PreCu, Precuneus; R, Right Hemisphere; RoMF, Rostral middle frontal; SF, Superior frontal; SP, Superior parietal; ST, Superior temporal; SM, Supramarginal; TrT, Transverse temporal.

^b^Importance ranking : The SVM feature importance ranking indicated that the SCEs were ranked based on their importance using the MI algorithm, with smaller values representing greater significance.

^c^N/A: not applicable.
