# Supplementary material for: Brain Structural Covariance Networks in Long-Term First-Person Shooter and Multiplayer Online Battle Arena Players: Cross-Sectional Study
Source: JMIR Serious Games. 2026 May 4;14:e79976. doi: 10.2196/79976 (PMC13150961; doi:10.2196/79976)
Supplement: Multimedia Appendix 3 [file games-v14-e79976-s003.docx]

**Table S1.** Nodes and nodal degrees of structural covariance edges correlated with weekly gaming hours in the first-person shooter group under the weight value>0.9.

| *P* value <.01 and weight value>0.9 | | | |
| --- | --- | --- | --- |
| Positive edges | | Negative edges | |
| Node^a^ | Degree | Node | Degree |
| L^b^ Or^c^ | 14 | L En^d^ | 4 |
| L TrT^e^ | 12 | R^f^ RoMF^g^ | 1 |
| R IT^h^ | 12 | L CMF^i^ | 1 |
| R IP^j^ | 12 | R FPol^k^ | 1 |
| R RoMF | 10 | L PerCa^l^ | 1 |
| L RoMF | 9 | N/A | N/A |
| R PoC^m^ | 9 | N/A | N/A |
| R SP^n^ | 7 | N/A | N/A |
| R SM^o^ | 6 | N/A | N/A |
| L PoC | 6 | N/A | N/A |
| L CMF | 5 | N/A | N/A |
| L ST^p^ | 5 | N/A | N/A |
| L PerCa | 5 | N/A | N/A |
| R CMF | 4 | N/A | N/A |
| R Op^q^ | 4 | N/A | N/A |
| L IP | 4 | N/A | N/A |
| R SF^r^ | 4 | N/A | N/A |
| L SM | 3 | N/A | N/A |
| L Cu^s^ | 3 | N/A | N/A |
| L SP | 3 | N/A | N/A |
| R FPol | 3 | N/A | N/A |
| L LO^t^ | 3 | N/A | N/A |
| L SF | 3 | N/A | N/A |
| R LO | 2 | N/A | N/A |
| R ST | 2 | N/A | N/A |
| R Lg^u^ | 2 | N/A | N/A |
| L Op | 1 | N/A | N/A |
| R Or | 1 | N/A | N/A |
| R MT^v^ | 1 | N/A | N/A |
| L PaH^w^ | 1 | N/A | N/A |
| L PreCu^x^ | 1 | N/A | N/A |
| L Ins^y^ | 1 | N/A | N/A |

^a^Node: Abbreviations correspond to cortical regions defined by the Desikan-Killiany atlas

^b^L: left hemisphere

^c^Or: pars orbitalis

^d^En: entorhinal

^e^TrT: transverse temporal

^f^R: right hemisphere

^g^RoMF: rostral middle frontal

^h^IT, inferior temporal

^l^CMF: caudal middle frontal

^j^IP: inferior parietal

^k^FPol: frontal pole

^l^PerCa: pericalcarine

^m^PoC: postcentral

^n^SP: superior parietal

^o^SM: supramarginal

^p^ST: superior temporal

^q^Op: pars opercularis

^r^SF: superior frontal

^s^Cu: cuneus

^t^LO: lateral occipital

^u^Lg: lingual

^v^MT: middle temporal

^w^PaH: parahippocampal

^x^PreCu: precuneus

^y^Ins: insula
